# NEWS – AGENCIES

**DOI:** 10.7189/jogh.08.010202

**Published:** 2018-06

## The Bill and Melinda Gates Foundation

➢ The BMGF announced that it will pay US$ 76 million of Nigeria’s debts, incurred when Nigeria borrowed from Japan in 2014 to fund its fight against polio. Nigeria reported no new polio cases in 2017 – in 2012, it has over 50% of all global polio cases – and the BMGF agreed to repay the loan after Nigeria achieved more than 80% vaccination coverage in at least one round each year in very high-risk areas across 80% of its local government areas. Bill Gates recently acknowledged the global progress towards eradicating polio – 30 years ago, there were 350 000 cases worldwide, compared to 20 in 2017. “The heroes who have made this progress possible are the millions of vaccinators who have gone door to door to immunise more than 2.5 billion children. Thanks to their work, 16 million people who would have been paralyzed are walking today,” he wrote in a recent blog. (CNBC, 18 January 2018)

➢ The BMGF is working with the Chinese Food and Drug Administration to help improve standards, and potentially posing a challenge to US and European regulatory dominance over pharmaceuticals. At a panel discussion at the World Economic forum in Davos, Mr Gates highlighted how regulatory dominance from the US and EU can lead to other agencies rejecting drugs that could have served a purpose in their own nations. He also pointed out that India and China cannot “afford to spend US$ 10,000 per citizen” for health care, so there is greater need for innovation, and will need to set their own medical rules on many fronts, including what makes a doctor, as training only one type of Western-style doctor will be too expensive. Mr Gates also spoke on the convergence on research between diseases such as cancer – mainly a problem in developed countries, and HIV and malnutrition, which are more prevalent in poorer nations. He spoke about research into the microbiome, which the BMGF funded to investigate malnutrition, and is now cited as the potential source of other diseases, including Alzheimer to autism. (Bloomberg, 25 January 2018)

➢ The Abraaj Group, based in Dubai, is reputedly one of the largest and most influential investors in the developing world, and manages nearly US$ 14 billion, including development agencies in the USA, Britain and France. Amongst others, the BMGF has US$ 100 million invested in Abraaj, and Bill Gates has previously called for private-sector investors to play a larger part in risky projects in the developing world. Both Mr Gates and Mr Jim Yong Kim, the World Bank president, have argued that it is possible for large pools of capital, such as private equity funds, insurance companies and pension funds – to earn large by profits by such investments. Abraaj holds a US$ 1 billion health fund, which aims to buy hospitals in Nigeria, India and Pakistan and improve their productivity – and profits. However, financial statements have shown US$ 200 million remains uninvested. The fund’s managers insisted that it needs cash on hand to buy hospitals, but has returned US$ 100 million. However, investors are following up with an audit, and are requesting forensic proof that Abraaj did not use the money to fund its own operations. When the allegations first arose, the World Bank began an investigation into corruption, according to correspondence seen by the *New York Times*. However, the review closed without finding evidence of wrong-doing. (New York Times, 2 February 2018)

➢ At the New England Journal of Medicine’s Shattuck Lecture, Bill Gates called for a co-ordinated global effort to combat major outbreaks of infectious diseases, noting that health infrastructure breaks down rapidly during outbreaks. He highlighted the need for early detection systems, better tools and response systems. Mr Gates also announced the launch of a US $12 million challenge (in conjunction with Larry Page, Google’s co-founder) to encourage scientists to work on a universal flu vaccine. “The world need to prepare for pandemics the way the military prepares for war,” said Mr Gates, and also called for a reserve corps of trained personnel and volunteers who would be on stand-by to fight spread of a pandemic; and for the US government to set up manufacturing and indemnification agreements and expedited review processes for drugs. (Forbes, 1 May 2018)

➢ Twelve African countries will meet in Cotonou, Benin, to discuss a joint response to the threat of Cassava Viral Disease (CVD), which is currently afflicting Africa. The high-level meeting, convened by the Government of Benin and supported by the Bill and Melinda Gates Foundation, and given the importance of cassava for Africa’s food security, a joint regional emergency response plan is needed urgently. The virus is devastating cassava crops in Eastern and Central Africa, and spreading to West Africa, and the meeting’s conclusions will be used towards sustainable food security in Africa. (The Guardian Nigeria, 3 June 2018)

## The GAVI Alliance

➢ Orange, the French telecommunications company, announced a US$ 5.47 million partnership with GAVI, to use its mobile technology to inform parents in Côte d'Ivoire about the importance of vaccinations. The targeted messages, sent as part of the M-Vaccin project, will remind parents about immunisation sessions, and their children’s schedule and dates. A 2015 study from the Expanded Program on Immunisation estimated that the M-Vaccin programme could reach more than 800 000 children in Côte d'Ivoire, and could be extended to other countries in West Africa. Orange will also provide an M-Vaccine app to health workers, enabling them to capture data from communities and populations they work with. The data can be used to create personalised immunisation schedules. The programme could have a significant impact on Côte d'Ivoire’s immunisation efforts, by contacting parents who live far from health centres or in hard-to-reach places. Dr Raymonde Goudou Coffie, Côte d'Ivoire’s Minister of Health and Public Hygiene, said “the Ministry of Public Health is working with partners like GAVI to keep those target populations better informed about the importance of vaccination. The solution, which will be made available to community health workers, will also make it possible to closely monitor pregnant women and children, and to reduce the number of people lost to follow-up.” (ITWeb Africa, 29 January 2018)

➢ GAVI has co-authored a study, published in *The Lancet*, which modelled the health and economic impact of vaccines for 10 diseases in 41 developing counties. The study estimated that vaccines administered from 2016 to 2030 could prevent 26 million deaths, and highlighted how sudden health care expenses force 150 million people into poverty each year. Indeed, this is one of the main factors that push people into extreme poverty – ie, living on less than US$ 1.90/d. Vaccines have the largest impact on reducing poverty arising from hepatitis B, helping 14 million people avoid medical impoverishment, and measles and meningitis A will also be significantly reduced by vaccines with an estimates 5 million and 3 million cases averted – and measles vaccination could prevent 22 million deaths. The study concluded that introducing vaccinations into the world’s poorest regions will have the most impact on reducing the number of deaths and the number of people forced into poverty by the cost of health care, and emphasises how ongoing investments in vaccinations could make a significant contribution towards the UN Sustainable Development Goals and universal health coverage. (Xinhua, 6 February 2018)

➢ Kenya's national rollout of a free cervical (Human Papillomavirus, or HPV) vaccine programme, due to launch in 2018, has been deferred to 2019. Dr Collins Tabu, head of Kenya's National Vaccines and Immunisation Programme, confirmed that this was due to high global demand for the vaccine, leading to manufacturers struggling to accommodate it. GAVI had requested the postponement to allow for more time to manufacture the vaccine. Currently, GAVI helps Kenya procure its vaccines through a co-financing model, where Kenya pays 10% of the vaccine costs, and GAVI pays the remaining 90%. Kenya piloted its HPV programmes 4 years ago among 20 000 school girls, and the update was 90%. In the programme’s first year, it will focus on girls aged 11-14 years, and in its second year, it will cover girls aged 10 years and under. HPV causes 99.7% of all cervical cancer cases worldwide, and is preventable and curable if detected early, but there is a shortage of effective screening and treatment in sub-Saharan Africa, contributing to a high number of cases and deaths. (Daily Nation, 17 February 2018)

➢ During a stakeholders’ meeting, Dr Seth Berkeley (GAVI’s CEO) disclosed that 800 000 unimmunised children died in Nigeria over the past 5 years, and that children are dying unnecessarily through a lack of routine immunisation. Shortfalls in Nigeria’s immunisation programmes have led to outbreaks of monkey-pox, Lassa fever, measles, meningitis and yellow fever. He called for Nigeria’s government and other stakeholders to work together to accelerate and strengthen routine immunisation, and that traditional rulers can help in ensuring immunisation, as they are close to the people. Faisal Shauib, the executive director of Nigeria’s National Primary Health Care Development Agency, said that the success in providing health care to people determined children’s futures, and tasked local governments and traditional rules to supervise and monitor the activities of primary health care facilities in their areas. (Premium Times, 8 March 2018)

➢ The new Ebola vaccination programme in the Democratic Republic of the Congo (DRC), funded by GAVI, must meet certain challenges to be successful, warns Dr Seth Berkley, GAVI’s CEO. First, response planners and vaccination teams must “hit the right notes” on several challenging communications messages – getting those messages wrong could endanger lives and undermine efforts to extinguish the outbreak. Second, the programme’s ring vaccination design can only succeed if vaccination teams have accurate list of people who should be vaccinated (eg, those at risk from Ebola due to contact with infected people, etc.). The vaccination effort got under way with health care workers being immunised in Mbandaka, in the region where the outbreak is taking place. There are 7500 vaccine doses on the ground, and the WHO has asked Merck – the manufacturer – to provide another 8600 doses. Dr Berkley warned that there is a danger that vaccines are seen as the “holy grail”, but the vaccination must be done in parallel with getting the epidemiology under control, uncovering contacts, isolation and safe burials. The Ebola vaccine was tested at the end of the 2013-16 outbreak in West Africa, and was found to be highly effective with no infections amongst immunised people, and gives hope that the DRC outbreak can be controlled. (STAT news, 23 May 2018)

## The World Bank

➢ The “Food for All” study from the World Bank and the Netherlands government shows that foodborne diseases costs India US$ 28 billion each year – equivalent to 0.5% of its annual gross domestic product. Indian people are moving from basic staples towards more nutritious food; whilst this could improve nutrition in India in the longer-term, currently it is introducing “risky foods” to peoples’ diets. According to one of the study’s researchers, ensuring food safety will reduce child mortality, and India should improve its food safety policy through co-ordination across the value chain, improve infrastructure, testing capacity, crop protection and animal health. Health ministers from different states across India adopted a joint resolution: “recognising that safe, hygienic and healthy diet is key to preventive healthcare which part of the first pillar of National Health Policy, the state health ministers agreed to improved convergence between National Health Mission and Safe and Nutritious Food campaigns,” according to a statement from India’s food regulator. The ministers agreed to support the development of robust food standards and codes of practice for food safety, improve regulation and food testing, and address micronutrient deficiencies through government schemes and promoting healthy diets. (Livemint, 9 January 2018)

➢ The World Bank has admitted that it repeatedly changed the methodology of its flagship “Doing Business” economic report, which ranks countries by the competitiveness of their business environments. Countries compete with each other to improve their rankings in the report, and it draws extensive media coverage. The World Bank’s Chief Economist, Paul Romer, confirmed that he would correct and recalculate the report’s rankings, going back at least four years. The revisions could significantly affect the rankings of several countries, including Chile, whose overall ranking had fluctuated between 25th and 57th since 2006. The World Bank’s corrections will focus on methodological changes that sharply penalised Chile under the recent term of its outgoing Socialist President, Michelle Bachelet. Mr Romer offered a personal apology to Chile, and to all countries adversely affected by the incorrect methodology, and said that he couldn’t defend “the integrity” of the process that led to the methodological changes. (Wall Street Journal, 12 January 2018)

➢ The World Bank is set for a US$ 13 billion capital increase, with the US dropping its objections to the Bank imposing measures that could reduce loans to China. The capital increase will be accompanied by other measures, such as operational changes and effectiveness reforms. The World Bank will increase its annual lending to about US$80 billion, from its current US$59 billion, with higher interest rates being charged to wealthier countries, including China. The World Bank will focus more on lending to “low middle-income countries,” and will gradually reduce lending to China, which it classifies as an “upper middle-income” country. However, China’s voting share in the World Bank will increase from 5.7% from 4.5%. The US had previously objected to the capital increase, arguing that the Bank does not need to lend significantly to China, given China’s economic growth and ability to raise money in financial markets. Other countries such as the UK had argued for capital increases, as the bank needs more firepower to finance projects. (Independent Ireland, 23 April 2018)

➢ According to the World Bank, global remittances reached a record US$ 613 billion in 2017, a 7% increase from US$ 517 billion in 2016. Following two years of decline, remittances to low- and middle-income countries rose by 8.5% to US$ 466 billion in 2017. Nigeria (US$ 22 billion), Egypt (US$ 20 billion) were the 5th and 6th top recipients, behind India (US$ 69 billion), China (US$ 64 billion), the Philippines (US$ 33 billion) and Mexico (US$ 31 billion). Sub-Saharan Africa saw an 11.4% increase in remittances to US$ 38 billion in 2017, underpinned by improved economic growth in developed countries and higher oil prices, with remittances to sub-Saharan Africa expected to grow by 7% to reach US$ 41 billion in 2018. However, it remains the most expensive place for money transfers, with average fees of 9.4% of the transaction’s value. (Forbes, 21 May 2018)

➢ The World Bank’s poverty assessment report for the Philippines shows that the country’s robust economic growth has helped to reduce the national poverty rate by 5% over the past 10 years, but that poverty remains high, and 22 million people still live below the poverty line. The report highlights that decline in poverty from 26.6% in 2006 to 21.5%, helped by the expansion of job opportunities outside agriculture, transfers from government social programmes, and remittances. However, economic growth in the Philippines is slower than many other East Asian countries, with less “pro-poor” policies. Poorer households are concentrated in the countryside and rely on agriculture for their main source of income – and many are located in areas prone to disaster or conflict, such as Mindanao. According to senior economist Xubei Luo at the World Bank’s Poverty and Equit Global Practice, increased investment in Mindanao to boost development would create opportunities for conflict-affected communities, improve services, and create more and better jobs. The government has prepared strategic plans to reduce poverty, which includes a target to reduce poverty to 13%-15% by 2022. (Strait Times, 30 May 2018)

## UN AIDS and the Global Fund

➢ According to the Namibia Network of AIDS Service Organisations (Nanasco), the Global Fund has allocated US$ 37 million to Namibia over the next three years, which is a “significant decrease” from the previous allocation of US$ 100 million. This reduction in funding has led to Nanasco reducing the number of Global Fund-financed projects to four. The funding decrease is due to Namibia achieving middle-income status, as the Global Fund priorities low-income countries with larger public health problems. Despite the cuts, Namibia has used funding to renovate clinics and health centres in six regions. Namibia may be eligible to re-apply for funding after 2020, depending on how it meets targets set under the dispersed grant. (The Namibian, 12 February 2018)

➢ The US is the largest contributor to the Global Fund, providing about US$ 1.35 billion a year, but President Trump’s proposed budget for 2019 contains a cut of US$ 425 million. The Health Global Access Project (Health GAP) estimates that there be an overall budget cut of US$ 1 billion from US global HIV programmes (including PEPFAR and the US President’s Malaria Initiative), and the Centers for Disease Control and Prevention. According to the Friends of the Global Fight Against AIDS, Tuberculosis and Malaria, the US$ 425 million cut in the Global Fund’s budget could result in 586 250 fewer lives saved, 454 750 fewer people on antiretroviral treatment, 131 750 fewer women on treatment to prevent mother-to-child transmission, amongst others. Asia Russell, Health GAP’s executive director said “if this budget passes as proposed, Donald Trump’s legacy will be millions of new and unnecessary infections and deaths – and a massive resurgence in the AIDS pandemic.” (Aidspan, 20 February 2018)

➢ Following on from other west and central African countries, The Gambia has launched its HIV/AIDS catch-up plan in the presence of Dr Djibril Daillo, the UNAIDS Regional Director for West and Central Africa, and Mrs Sirra Horeja Ndow, UNAIDS Country Office Director. The plan was developed following the UN High-Level meetings on Ending AIDS in 2016. The Gambia’s catch-up plan was developed through a participatory process supported by UNAIDS, and its total cost is US$ 22 million, with an estimated funding gap of US$ 18 million – which is essential to ensure UNAIDS 90-90-90 goal (90% of people living with HIV know their status, 90% of people receiving treatment, and 90% of people have viral suppression). However, The Gambia’s political transition to fuller democracy is an opportunity to galvanise its HIV response, and for policy shifts that will enable The Gambia to tackle HIV, and to mobilise resources. (The Point, 5 March 2018)

➢ In a policy shift, the Global Fund will no longer allow countries to apply for extensions to use up any remaining grant funds. Previously, the Global Fund was more flexible, allowing unspent funds to be carried forward, but after the current funding implementation cycle, all unspent funds must be returned to the Global Fund for re-allocation. Some developing countries can find it challenging to spend funds on agreed programmes, as they lack institutional capacity to reach high- and low-density areas in the countries with medical services, and there may also be gaps in technical knowledge. The Global Fund considers each country’s ability to spend funds during the implementation period – known as the absorption rate – when determining each country’s funding allocation. It is working with countries to improve grant and project management, including technical and administrative support, and fostering partnerships to examine barriers towards programme implementation. (Devex, 7 March 2018)

➢ The lives of the hundreds of thousands of HIV-positive people in Uganda are endangered as the country’s supplies of the antibiotic Septrin (given to people on antiretroviral treatment to fight infections) are running out. Sarah Achieng Opendi, Uganda’s state minister for health, said that the country experienced a funding gap, and could procure Septrin in time – most of the public-health facilities and ART-accredited sites have not received Septrin in time. The government is expected to receive money from the Global Fund in July, when further supplies are expected. There are concerns that Septrin shortagles will weaken immunity amongst people with HIV. Septrin is also an important treatment for conditions in non-HIV positive people, and is a low-cost and accessible drug than can be produced in-country so imports are not required. (The Guardian, 22 May 2018)

## United Nations

➢ Despite the budget of the UN’s World Food Programme (WFP) being at a record high, it is insufficient to support the number of refugees and people in crisis situations who need emergency food aid. In 2017, it received US$ 9.1 billion to operate, but receives US$ 6.8 billion, thanks to ongoing conflicts in countries such as Syria and Yemen, and other crises. Faced with these shortfalls, the WFP has made cutbacks to the number of people it supports, eg, suspending food aid to Somalia for 500 000 people in December 2017; and in Syria, it provided food aid to 2.8 million people in January 2018 – down from 4 million people in 2017. Other cost-saving strategies include cutting the number of calories in food aid. A daily WFP full ration would contain 2100 calories – the minimum for adults – but in Yemen, the ongoing difficulties of providing food aid has led to 40% calorie cuts being imposed on half of Yemen’s food aid. As the WFP is normally the “provider of last resort”, there is no guarantee that other agencies can fill these gaps. If there are deep or sustained cuts to food aid, refugees face an increased risk of malnutrition and other health problems. Food aid can even become a medium of exchange in refugee camps, with parents reducing their food intake to ensure that they can feed their children, or pay for their children’s health care or education. (NPR, 10 January 2018)

➢ In its latest review of least-developed countries (LDC), the United Nations (UN) recommended that two Pacific Island states – the Solomon Islands and Kiribati – be removed from the list. The LDCs comprise the world’s poorer and weaker states, and countries in this category receive additional international support with trade and developmental assistance. LDC status is assessed according to health and education, economic vulnerability, and *per capita* national income. Both these nations have recorded enough gains across these categories to graduate out of LDC status, leaving Vanuatu and Tuvalu as the only remaining Pacific Island states in this category. Despite the loss of LDC status, the Solomon Islands and Kiribati will continue their development partnerships with the World Bank and the Asian Development Bank. However, both states are extremely vulnerable to the effects of climate change and extreme weather activity, and both retain funding from the UN’s Green Climate Fund that favours Small Island Developing States. (The Diplomat, 23 March 2018)

➢ Mark Lowcock, the UN emergency relief co-ordinator, has urged the Saudi-led military coalition, which controls Yemen’s ports, to expedite the imports of vital food and fuel supplies. Mr Lowcock warns that a further 10 million Yemeni people could face starvation by the end of 2018 – after three years of war, Yemen is the world’s worst humanitarian crisis, with 8.4 million people already severely short of food and at risk of starvation. Yemen has traditionally imported 90% of its food supplies, and for several weeks at the end of 2017, the Saudi coalition imposed a blockade on Yemeni ports, to prevent weapons imports. This had a serious impact on food and fuel imports, which remain well short of pre-blockage levels – although the blockade has been lifted following international pressure, ship inspections have been tightened. The UN announced in March 2018 that it would beef up its own inspection of ships, in efforts to speed up the delivery of aid to Yemen. Mr Lowcock also noted that key supplies, including some needed to combat cholera, remain on the prohibited list of imports. (Reuters, 1 April 2018)

➢ In an effort to pressurise all side to end South Sudan’s 5-year conflict, the UN has backed a decision to renew sanctions on the country’s leaders, and to consider imposing asset freezes and travel bans. The UN Security Council voted on 31 May to renew some sanction on South Sudan, and to consider imposing travel bans and asset freezes on six South Sudanese leaders if the conflict did not stop by 30 June. Seven million people rely on humanitarian aid to stay alive, and Mark Lowcock, the UN emergency relief co-ordinator, said that its aid workers would remain, despite kidnappings, murders and looting. South Sudan has been afflicted by civil war since 2013, when troops loyal to President Salva Kiir clashed with the troops of the sacked vice president, Riek Machar. 1.76 million people are internally displaced, and 2 million people have fled to neighbouring countries. The May 2018 round of peace talks failed, and the situation continues to deteriorate, with a collapsed economy and scorched earth tactics - including murder and rape – and some areas are close to the brink of famine. (Reuters, 4 June 2018)

➢ The UN General Assembly has called for more action to end tuberculosis (TB) by 2030, noting that it is not on track to meet this particular Sustainable Development Goal, with inadequate progress, action and investment. There was a funding gap of US$ 2.3 billion in 2017, and multi-drug resistant is an emerging threat. TB is treatable, but kills more than 4500 people each day, and globally more than 50% of cases are undiagnosed. Miroslav Lajcak, the President of the UN General Assembly, stated that TB will cost the global economy US$ 1 trillion by 2030. The UN will hold its first-ever high-level meeting on the fight against TB in September, and Mr Lajcak urged the General Assembly to galvanise the global momentum to end TB. The UN Secretary General, Antonio Guterres, calls for this meeting to ensure “global rhetoric is translated to local actions in communities.” (Xinhau Net, 4 June 2018)

## UNICEF

➢ According to a UNICEF report, Egypt’s poverty rates have increased over the past 15 years, to reach 27.8% in 2018, leading to 10 million children being “multi-dimensionally poor” (ie, facing risks of stunting, lacking access to education, clean drinking water, health care, and potential violence). Poverty rates have increased in Egypt, since President Abdel Fattah Al-Sisi took office in 2013 following a military coup, and political upheaval, air crashes, and security fears have all devastated the country’s crucially-important tourism industry. The government has also tightened up on the activities of NGOs, banning them from engaging in rights works or anything that could be said to harm national security – making it difficult for NGOs to deliver services. Moreover, a report published in *Al-Monitor* found that 60% of children in the Manshiyat Nasr neighbourhood of Cairo remained outside the formal education system, and are therefore unable to access government allowances that rest on children attending school. (Middle East Monitor, 10 January 2018)

➢ According to UNICEF, nearly 30% of young people - aged 15-19 years - who live in countries affected by conflict or natural disaster are illiterate. This problem - affecting nearly 60 million young people worldwide – is more pronounced amongst girls compared to boys, with 33% of such girls do not have basic literacy skills, compared to 24% of boys. UNICEF calls for more funding for education, particularly during humanitarian crises, stating that only 3.6% of humanitarian funding is allocated for young people's education, making it one of the least funded humanitarian sectors. Countries with the highest rates of illiteracy amongst young people include Niger (76%), Chad (69%), South Sudan (68%) and the Central African Republic (64%). UNICEF has appealed for US$ 900 million for countries ravaged by man-made and natural disasters, to be used on accelerated learning, teacher training, and school refurbishment and supplies. UNICEF also called for governments to provide more early learning, and to provide young people who are illiterate with specially-designed alternative education programmes. (Voice of America, 31 January 2018)

➢ UNICEF has called on the Gombe State Government to expand the treatment of acute malnutrition in the Nigerian state to cover all local government areas in Gombe, up from the present three areas. The Community-based Management of Acute Malnutriton (CMAM) is being implemented in three local government areas which include Nafada, Gombe and Dukku, and very recently, Kaltungo. UNICEF’s Drissa Yeo has called on Gombe’s governor, Ibrahim Hassan Kankwambo, to order to the release of N248 million (US$ 690 000) support for the programme, and expand it across Gombe State. UNICEF has also called for the Gombe State Government to implement the Female Teachers’ Training Scholarship Scheme (FTTSS) by endorsing a memorandum of understanding to sponsor more girls, and enunciate a policy on recruiting and employing more women teachers and FTTSS graduates to teach in rural schools. (This Day, 12 February 2018)

➢ UNICEF’s latest report on global newborn mortality rates – *Every Child Alive* – shows that global deaths of newborn babies remain alarmingly high, particularly in low-income countries, with Pakistan, the Central African Republic and Afghanistan being the worst-affected. In Pakistan, 1-in 22 babies die, in the Central African Republic 1-in-24 babies die, and 1-in-25 die in Afghanistan. In comparison, just 1-in-1111 babies die in Japan, one of the countries with the lowest mortality rate for newborns. Across all low- and middle-income countries, the average newborn mortality rate is 27 deaths per 1000 births, and in high-income countries the rate is 3 deaths per 1000 births. UNICEF stated that if every country reduced its newborn mortality rate to the levels in high-income countries, by 2030, 16 million lives could be saved – but many of these preventable deaths arise from shortages of health-workers and midwives. “We know that we can save the vast majority of these with affordable, quality health care solutions for every mother and every newborn. Just a few small steps from all of us can help ensure the first small steps of each of these young lives,” said Henrietta Ford, UNICEF’s Executive Director. (The Journal Ireland, 20 February 2018)

➢ UNICEF has warned that hundreds of thousands of children in the Democratic Republic of the Congo (DRC) face imminent death from hunger. UNICEF said that deaths in the Kasai region of the DRC could “skyrocket” without urgent humanitarian assistance – violence erupted in Kasai in August 2016, displacing 1 million people from their homes. According to UNICEF, 770 000 of the region’s children are malnourished, with 400 000 at risk of death, and UNICEF has appealed for US$ 88 million to support these children with 50% of funds earmarked for child nutrition. A lull in violence has led to more people returning to their communities, making it easier to provide humanitarian assistance and to treat malnourished children. Beyond the immediate threats from violence and malnourishment, Kesai faces an ongoing health crisis, including measles outbreaks. During the violence, 200 health centres were looted, burned or destroyed, along with 100 schools; and another 300 schools were used for military purposes. (The Guardian, 11 May 2018)

## World Health Organization (WHO)

➢ Ghana is working with the WHO to confirm the elimination of trachoma as a public health problem, making it the 6^th^ country to officially eliminate the disease. Trachoma is a contagious eye infection that can cause scarring of the inner eyelid and irreversible blindness. Ghana’s progress on trachoma is part of the African Leaders’ Malaria Alliance (ALMA) annual scorecard on disease progress, published at the 30th African Union Heads of State summit. For the first time, the scorecard revealed countries’ progress on neglected tropical diseases (NTD), and Ghana shows major progress, ranking 6th out of 47 countries in reaching in people in need of mass NTD treatment. The scorecard is reviewed by African heads of state each year, and NTDs, alongside malaria, and maternal and child health, are top health priorities for the continent. The scorecard was developed by the WHO in collaboration with United to Combat NTDs, and tracks progress for the 47 NTD-affected countries on the strategies to prevent and treat the five most common NTDs (lymphatic filariasis, onchocerciasis, schistosomiasis, soil-transmitted helminths and trachoma). (Business Ghana, 29 January 2018)

➢ In 2013, the WHO committed to eradicating yaws – a disfiguring skin disease which affects more than 64 000 people each year – by 2020. This decision was spurred on by the discovery that yaws can be easily treated by the antibiotic azithromycin, at an estimated cost of US$ 100 million. However, there are concerns that this could fail, since the recent discovery that wild primates can harbor the bacterium (*Treponema pallidum* subsp *pertenue*) that causes the yaws infection. Epidemiologists admit that the WHO has not yet addressed the potential threat posed by wild primate populations to the elimination of yaws – although it is unknown if yaws can be transmitted from primates to humans, it would be “remiss” not to pay attention to it. A similar problem is faced by the campaign to eradicate Guinea worm disease, when it was discovered that dogs and other animals can carry the parasite. Additional investments were then required for public education, extra monitoring and treatment, and the disease remains uneradicated. (Nature, 8 February 2018)

➢ South Sudan’s largest cholera outbreak began more than 18 months ago – which infected more than 20 000 people and killed 436 – is now officially declared over. The outbreak occurred in mid-2016, as fresh fighting began in the city of Juba, and spread to 26 counties across the country. No new cases have been reported in the past 7 weeks, although complacency must be avoided. The outbreak took hold as renewed fighting made it difficult for people to attend health facilities, many NGO health care workers were evacuated, and the movement of people helped to further spread the disease. The WHO and health officials are advising people to drink only clean water, use boiled water or water treated with chlorine for other household uses, and to always wash both hands with soap after using the latrine or before handling food; and to avoid defecation near any source of water. (Voice of America, 8 February 2018)

➢ The WHO is meeting to discuss whether to declare a “public health emergency of international concern” over the risk of Ebola spreading beyond the Democratic Republic of Congo (DRC). To date, 45 people are believed to have been infected in the current Ebola outbreak, and 25 deaths are being investigated. Ebola emerged in rural areas in DRC, but there is one confirmed case in Mbandaka, a major transport hub and home to 1 million people, which could lead to an “explosive increase” in cases. Mbandaka’s location on the River Congo raises the possibility of Ebola spreading to Congo Brazzaville and the Central African Republic, as well as DRC’s capital, Kinshasa, which has a population of 10 million. Isolation and management facilities have been set in Mbandaka, and more than 4000 doses of an experimental vaccine sent by WHO has arrived in Kinshasa; with priority for immunisation given to those who mayhave been in contact with people suspected of being infected with Ebola. The WHO has already admitted that its response to the 2014-16 Ebola outbreak – which killed more than 11 000 people – was too slow. (BBC, 18 May 2018)

➢ Globally, 90% of people who need assistive technology (eg, glasses, wheelchairs or hearing aids), cannot access it, and this is particularly acute in developing countries. To address this, the World Health Assembly passed a draft resolution to improve access, and the WHO was actioned to report by 2021 on effective access to assistive technology; and to provide technical and capacity-building support to countries. According to the WHO, 70 million people world-wide need a wheelchair, but only 5-15% have access to one, hearing aid production meets only 10% of global need, and 200 million people with visual problems do not have access to glasses or other devices. At the World Health Assembly, many countries, including Bhutan, highlighted how lack of access affects the most vulnerable groups. Ageing populations is one of the factors lying behind the increasing need for assistive technology, but some countries that have experienced conflict also need assistive technologies for younger people. (Intellectual Property Watch, 26 May 2018)

